# Supplementary material for: MicroRNA-34a dependent regulation of AXL controls the activation of dendritic cells in inflammatory arthritis
Source: Nat Commun. 2017 Jun 22;8:15877. doi: 10.1038/ncomms15877 (PMC5489689; doi:10.1038/ncomms15877)
Supplement: Supplementary Information [file ncomms15877-s1.pdf]

Type of file: pdf
Title of file for HTML: Supplementary Information
Description: Supplementary Figures and Supplementary Tables

Type of file: pdf
Title of file for HTML: Peer Review File
Description:

Supplementary Table 1. Demographic and clinical information of Cohort 1

**Subjects**	**Age** mean ± SD	**Disease duration** mean ± SD	**DAS28 ESR** mean ± SD
RA patients (n=30)	57.9±13.8	10.42±14.02	3.47±1.4 (16% HDA 40% MDA 44% LDA)
Healthy donors (n=19)	47.7±11	N/A	N/A

Peripheral blood CD1c^{+} dendritic cells were isolated from these patients and expression of microRNA34a and *AXL* analyzed by qPCR. RA, Rheumatoid Arthritis; DAS28, Disease activity Score: DAS28 ≤ 3.2 = low disease activity (LDA)/in remission; DAS28 >3.2 ≤5.1 = moderate disease activity (MDA); and DAS28 >5.1 = high disease activity (HAD). DAS28 was calculated using ESR value (Erythrocyte Sedimentation Rate).

Supplementary Table 2. Demographic and clinical information of Cohort 2

**Subjects/tissue**	**Age** mean ± s.d.	**Disease duration (months)** mean ± s.d.	**DAS28 ESR** mean ± s.d.
**All RA patients/Blood (n=19):**	58.1 ± 14.4	29.7 ± 27.1	5.07 ± 0.68
naïve to DMARDs (n=7)	62.1 ± 9.9	6.9 ± 3.8	4.97 ± 0.65
resistant to MTX (n=9)	54.2 ± 17.7	32.0 ± 17.0	5.06 ± 0.66
resistant to MTX and TNF inhibitors (n=3)	60.0 ± 11.1	76.0 ± 18.3	4.88 ± 0.36
**RA patients/synovial biopsies** (n=9)	57.1 ± 14.4	31.3 ± 18.9	5.37 ± 0.65
**PsA/Blood** (n=16)	58.0 ± 14.1	N/A	3.41 ± 1.28
**PsA/Synovial biopsies** (n=5)	58.7 ± 4.0	22.0 ± 15.1	4.32 ± 0.43
**Healthy donors/Blood** (n=11)	51.5 ± 8.8	N/A	N/A

$CD1c^+$ dendritic cells were isolated from these patients and microRNA34 and *AXL* analyzed by qPCR. RA, Rheumatoid Arthritis; DAS28, Disease activity Score: DAS28 ≤ 3.2 = low disease activity (LDA)/in remission; DAS28 >3.2 ≤5.1 = moderate disease activity (MDA); and DAS28 >5.1 = high disease activity (HAD). DAS28 was calculated using ESR value (Erythrocyte Sedimentation Rate). Disease modifying anti-rheumatic drugs, DMARDs that include methotrexate (MTX). TNFi, biologic treatment: TNF inhibitors.

Supplementary Table 3. Demographic and clinical information of Cohort 3

	**Healthy donors**	**RA Naïve to treatment**	**RA DMARDs resistant/biologic therapy resistant**	**RA in remission**	**OA**
Numbers	10	20	20	10	10
Age (median and range)	51 (34-70)	56.5 (20-81)	52.5 (25-74)	56.6 (45-72)	60.5 (50-83)
Disease duration years (median and range)	N/A	1.5 (0.3-3)	5.85 (0.5-20)	17 (6-28)	N/A
DAS28-ESR (mean ± s.d)	N/A	4.64 ± 1.26	5.12 ± 1.11	1.89 ± 0.54	N/A

Soluble AXL and GAS6 were measured in sera of these patients. Disease modifying anti-rheumatic drugs, DMARDs; osteoarthritis, OA. Not available N/A. See legend for Supplementary Table 1 for more details. RA Naïve to treatment = RA patients naïve to any Disease-Modifying Anti-Rheumatic Drugs (DMARDs); RA in remission = RA patients in stable remission achieved under a combination therapy of Methotrexate + TNF inhibitor; RA resistant to treatment = RA patients not responding to conventional DMARDs or a combination of Methotrexate plus TNF inhibitors.

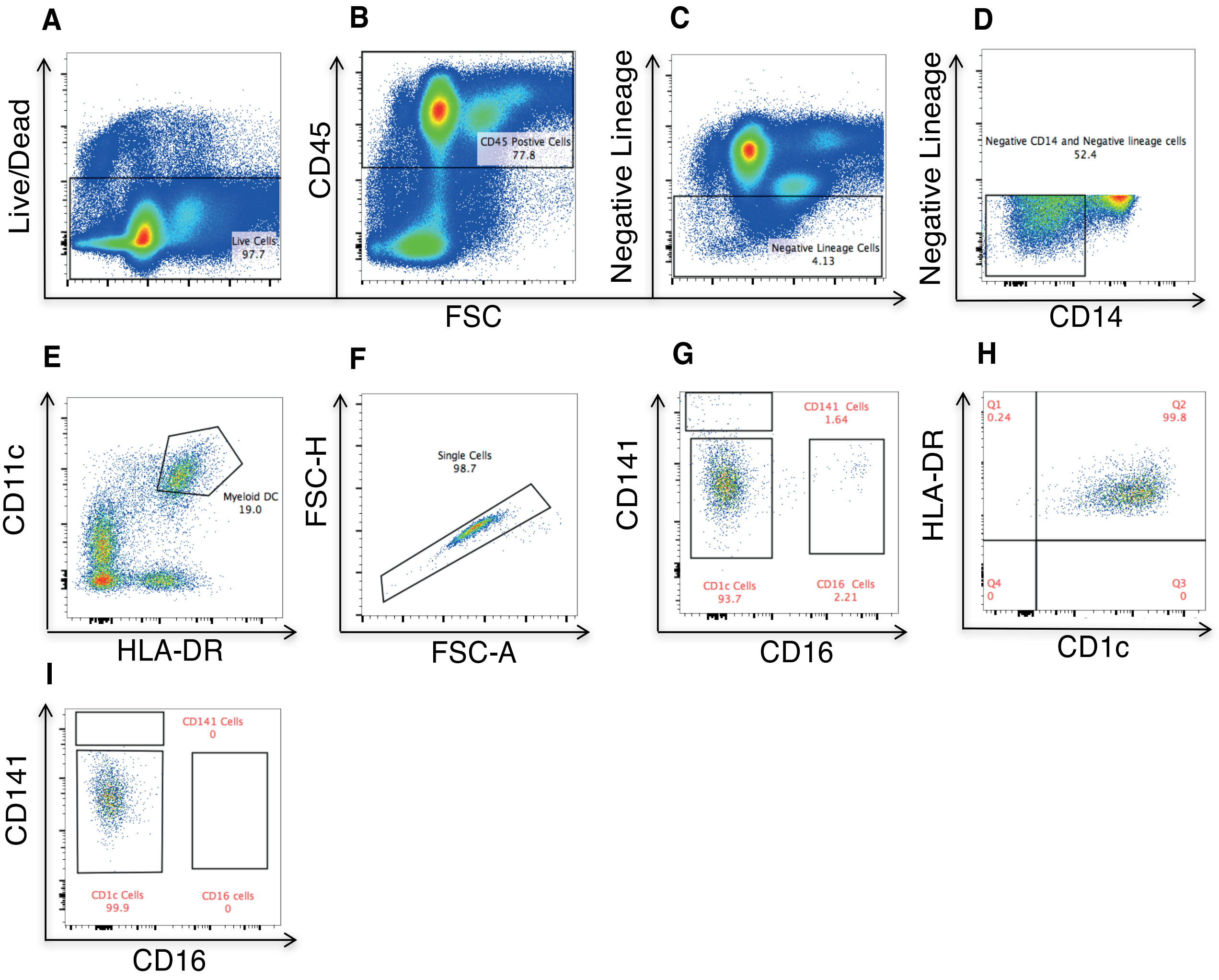

Supplementary Figure 1. Gating strategy for sorting CD1c$^+$ dendritic cells from blood and synovial tissues. A representative stepwise gating strategy for sorting human DC subsets is shown. (A) Live cells were gated, (B) CD45 positive cells were gated, (C) lineage positive for CD56, CD3, CD19, CD15 and CD117 were excluded, (D) CD14$^+$ monocytes were excluded, (E) HLA-DR^{++} CD11c^{++} high conventional DCs were gated, (F) DC doublets were excluded, (G) CD141$^-$ CD16$^-$ DC were gated, which showed CD1c$^+$ positivity (H). After sorting, CD1c$^+$ cells were recalled verifying the purity of sorting (I). The antibodies used in the sorting are described in the Methods section.

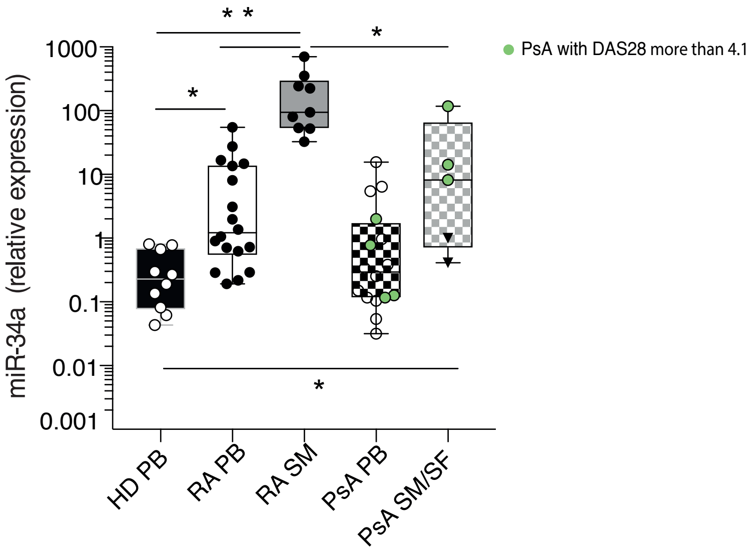

Supplementary Figure 2. MiR-34a expression is up-regulated in synovial tissue CD1c$^+$ DCs but not blood DCs of Psoriatic Arthritis (PsA) patients. The miR-34a expression in DCs from peripheral blood **(**PB) of healthy donors (HD) and PB and synovial membrane (SM) from RA patients are as in Figure 1 and used for comparison. In addition, CD1c$^+$ DCs were sorted from PsA PB (n=16), PsA synovial membrane (SM, n=3) and PsA synovial fluid (SF, n=2; marked by triangle). The expression of miR-34a was reanalysed by qPCR in all samples at once. **$p<0.01$ and *$p<0.05$ Kruskal Wallis test with Dunn's multiple comparison test. Data are presented as median and inter-quartile range; each dot represents a single sample. The green PsA dots represent patients with DAS>4.1. Demographic and clinical information for the HD and patients are presented in Supplementary Table 2.

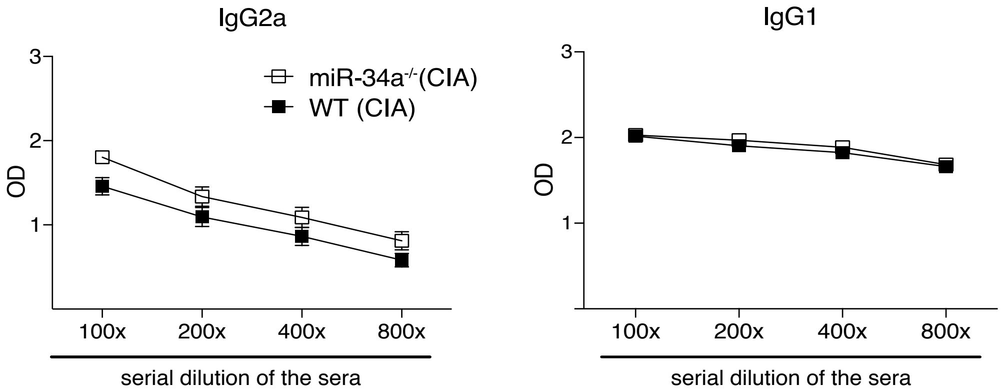

Supplementary Figure 3. MiR34a$^{-/-}$ mice show no difference in anti-collagen antibody production compared to WT mice. WT (n=15) and miR-34a$^{-/-}$ (n=14) mice were sensitized according to the protocol described in Methods. Mice were monitored for disease onset and paw swelling from day 10. Mice were culled on day 33 and serum harvested. The titres of anti-collagen IgG1 and IgG2a antibodies were evaluated with specific biotin-labelled anti-IgG1 and anti-IgG2a antibodies. Sera were serially diluted as indicated on the graph. Two-way Anova test demonstrated no difference in the antibody response between the genotypes.

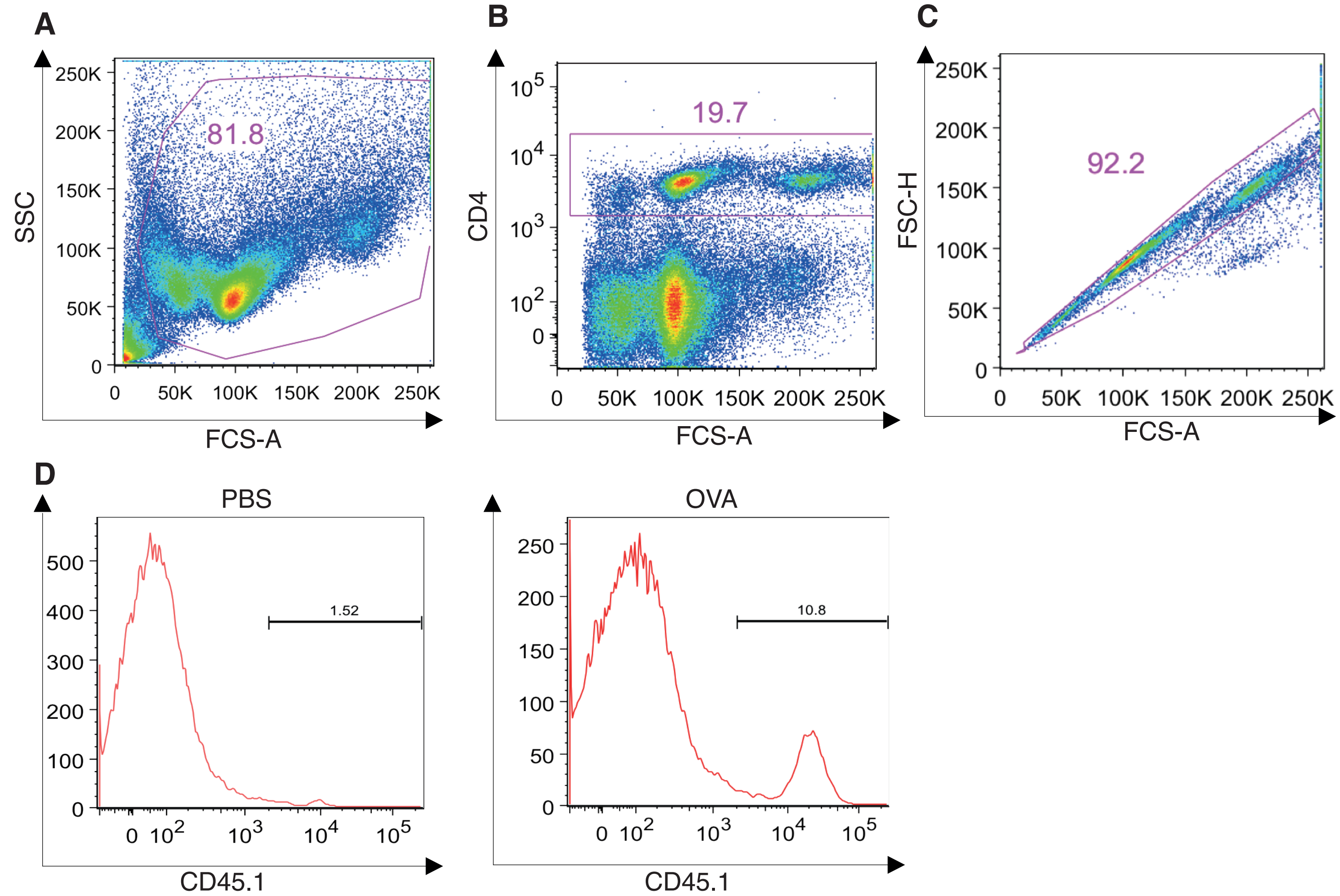

Supplementary Figure 4. Gating strategy for OT-II CD4⁺ CD45.1⁺ cells in recipient mice. (A-D) OT-II CD4⁺ CD45.1⁺ cells were injected into recipient mice WT or miR-34a$^{-/-}$ mice intravenously. After 24h OVA peptide or PBS was injected intramuscularly as described in Materials. Five days later, draining lymph nodes were isolated and total CD4⁺ gated (A-B) followed by exclusion of doublets (C) and gating OT-II CD45.1⁺ CD4⁺ donor cells (D), which show expansion of OT-II CD45.1⁺ CD4⁺cells in recipients receiving antigen but not in recipients receiving PBS.

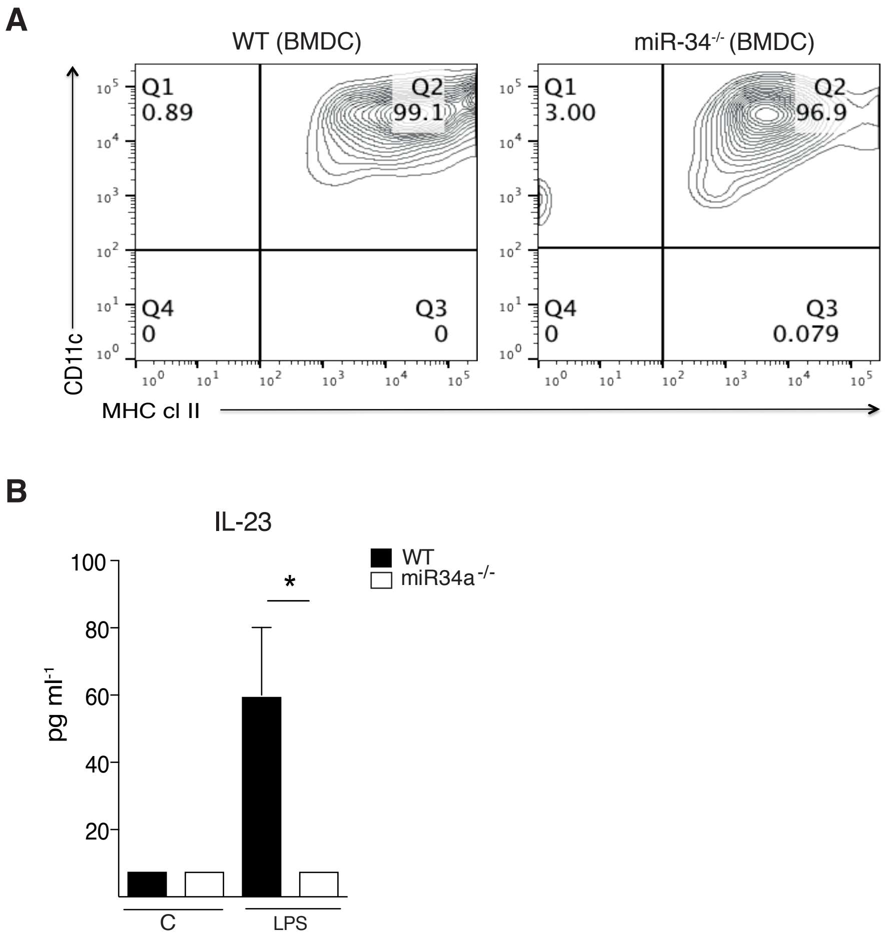

Supplementary Figure 5. MiR-34a$^{-/-}$ DCs show reduced production of IL-23 upon stimulation. (A-B) Bone marrow cells from WT and miR34a$^{-/-}$ mice (n=3, pooled) were cultured with GM-CSF (20 ng/ml). After 3 and 6 days the medium was replaced with fresh medium supplemented with GM-CSF. At day 8, the purity of the DCs was evaluated by FACS (A) and then cells were stimulated with LPS (1 ng/ml) for 48h (B). (A) A representative FACS dot plot for MHC class II and CD11c shows that there was no difference in the DC differentiation process between miR-34a$^{-/-}$ and WT bone marrow. (B) IL-23 (p19/p40) enzyme-immunoassay on un-stimulated and stimulated supernatants demonstrated significantly reduced production of IL-23 by LPS-stimulated miR-34a$^{-/-}$ derived DCs. Data are presented as mean ± s.e.m. of 3-4 replicates. *$p<0.05$ Kruskal Wallis test and Dunn's multiple comparison test

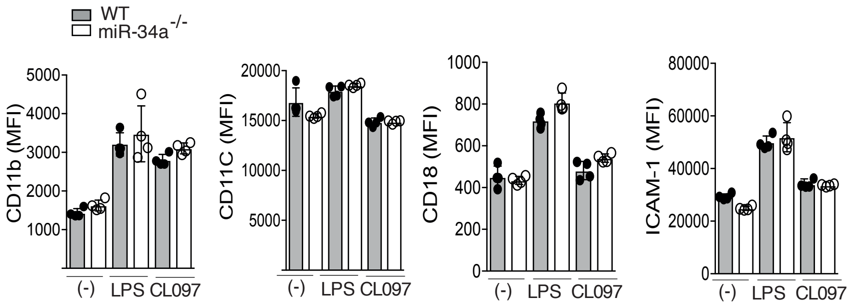

Supplementary Figure 6. MiR-34a$^{-/-}$ DCs show similar expression of adhesion molecules as WT DCs. Bone marrow cells from WT and miR34a$^{-/-}$ mice (n=3 pooled) were cultured with GM-CSF (20 ng/ml). After 3 and 6 days, the medium was replaced with fresh medium supplemented with GM-CSF. At day 8, the purity of DCs was evaluated by FACS (as shown in sup. Fig. 5A) and cells were stimulated with LPS (1 ng/ml) or CL097 (1 µg/ml) for 48h and the expression of adhesion molecules CD11b, CD11c, CD18 and ICAM-1 evaluated by flow cytometry mean fluorescent index (MFI) was no different between the genotypes. Data are presented as mean ± s.e.m of 4-8 replicates from 2 independent experiments; *$p<0.05$, Kruskal Wallis test and Dunn's multiple comparison test.

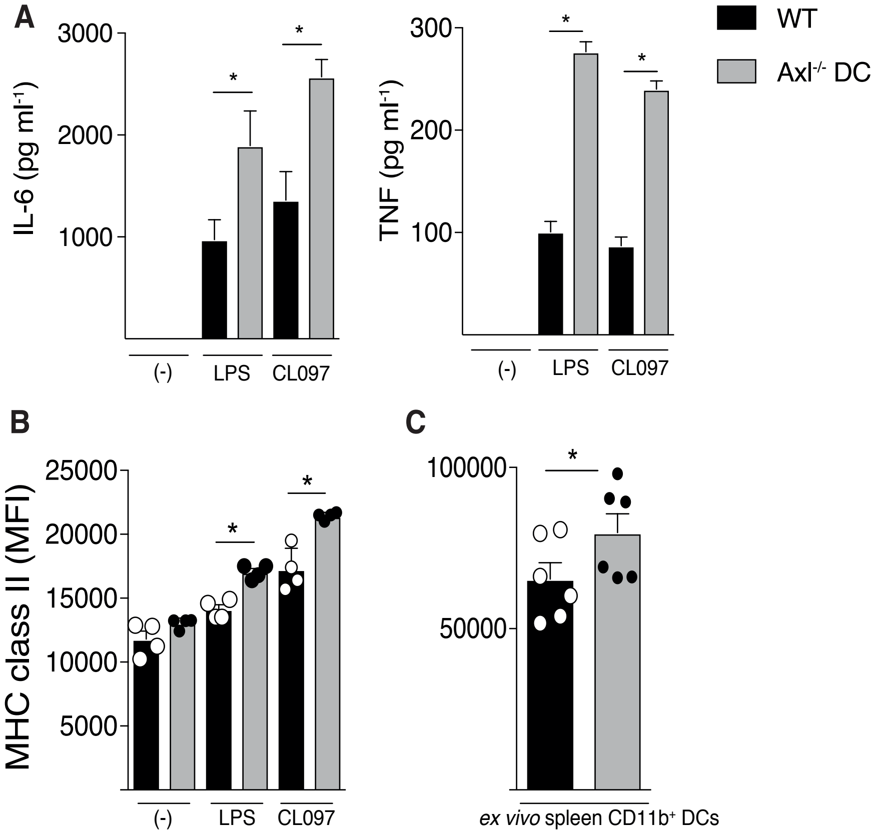

Supplementary Figure 7. Axl$^{-/-}$ DCs show enhanced production of cytokines and expression of MHC class II. (A-B) Bone marrow cells from WT and Axl$^{-/-}$ mice (n=3, pooled) were cultured with GM-CSF (20 ng/ml). After 3 and 6 days, the medium was replaced with fresh medium supplemented with GM-CSF. At day 8, cells were stimulated with LPS (1 ng/ml) or CL097 (1 µg/ml) for 48h; and supernatant TNF and IL-6, quantified by enzyme-immunoassay, was significant increased in Axl$^{-/-}$ DC cultures (A), and surface expression of MHC class II quantified by flow cytometry mean fluorescent index (MFI) was significant increased in Axl$^{-/-}$ DCs (B). **(C)** Expression of MHC class II quantified by flow cytometry mean fluorescent index (MFI) was constitutively significantly increased *ex vivo* on spleen Axl$^{-/-}$ CD11b$^+$CD4$^+$ DCs. Data are presented as mean ± s.e.m of 4-8 replicates from 2 independent experiments *$p<0.05$ Kruskal Wallis test and Dunn's multiple comparison test.

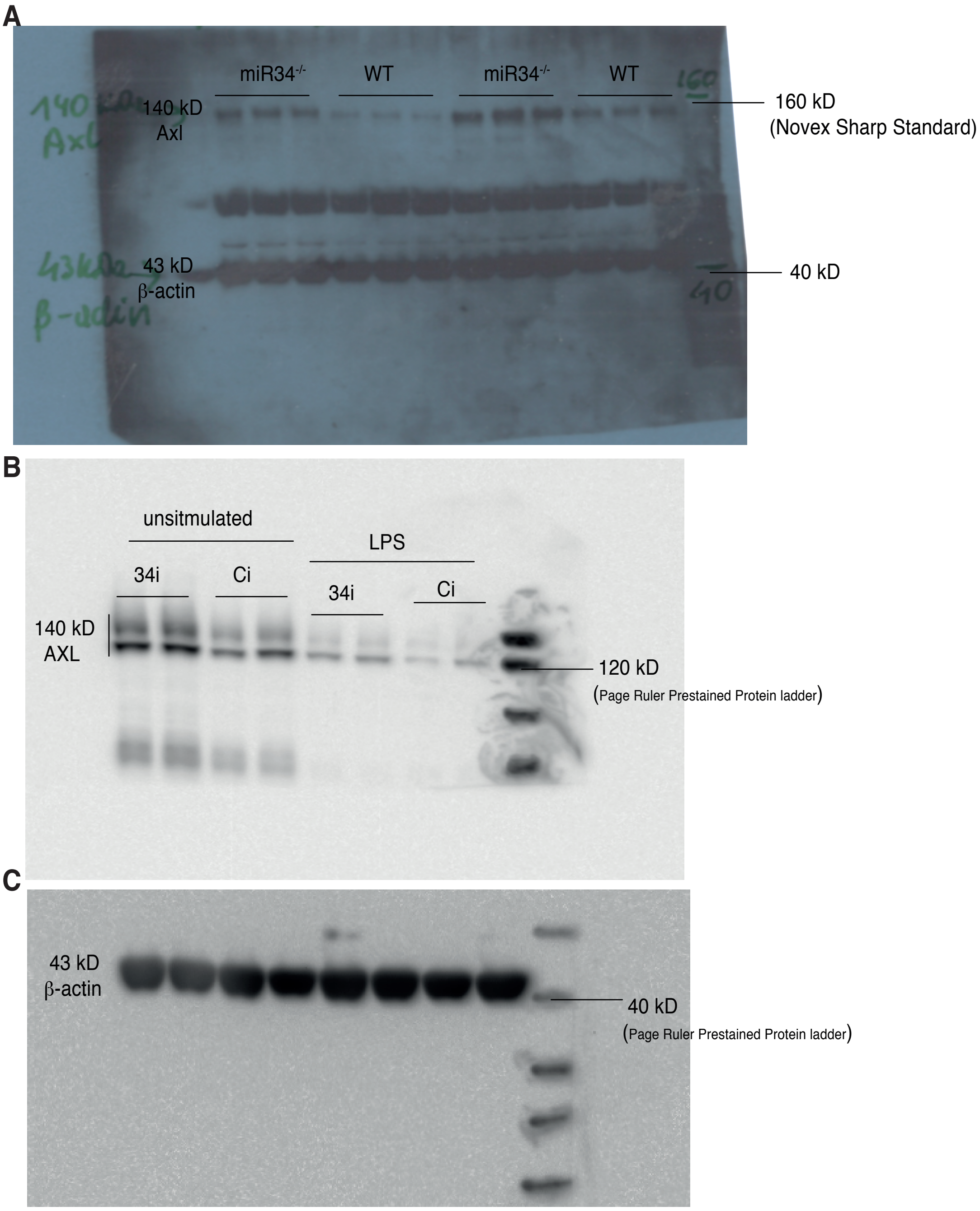

Supplementary Figure 8. miR-34a targets AXL. (A) GM-CSF-differentiated mouse miR-34a$^{-/-}$ DCs contain higher concentrations of total AXL protein compared to WT as demonstrated by Western Blot. Data presented on the blot were generated in 2 experiments; and each lane represents one biological replicate. The blot was developed with anti-Axl antibody, stripped and developed with anti β-actin antibody. Details on the antibodies are provided in Method section. The film was developed with Super Signal West Pico Chemiluminescent kit (#34080/ThermoFisher Scientific) in Kodak X-OMAT 1000. Novex Sharp Protein standard (#LC5800/Life Technologies) was used to estimate the size of the bands. (B-C) Healthy donor human PB monocyte-derived DCs transfected with miR-34a inhibitor (34ai) and stimulated with LPS (1 ng/ml) or left un-stimulated showed higher expression levels of AXL compared with cells transfected with control inhibitor (Ci). The Page Ruler Pre-Stained Protein ladder (#26616/ThermoFisher Scientific) was used to estimate the size of the bands. The blot was cut in half at the level of 70kDa marker; and the upper half was incubated with anti-AXL antibody while a bottom half was incubated with and β-actin antibody (details in Material sections). Each lane represents one biological replicate. Blots were developed with the Chemiluminescent kit as above and imaged with Azure C500 imaging system (Azure Biosystems).

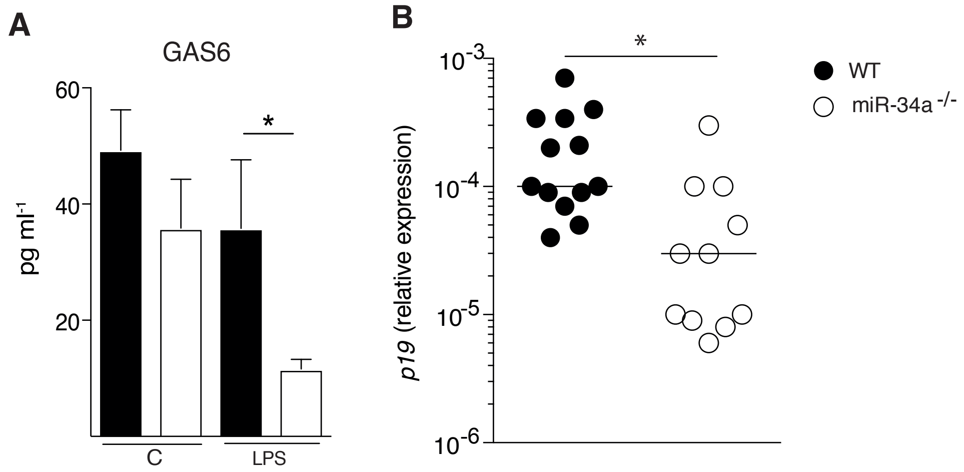

Supplementary Figure 9. GAS6 production and *p19* expression levels in miR-34a$^{-/-}$ dendritic cells and mice. (A) Supernatant from miR-34a$^{-/-}$ DC culture (as in Sup Fig. 5B) contained lower concentrations of GAS6 upon LPS stimulation as compared to similarly stimulated WT cells. Data are presented as mean ± s.e.m of 8 replicates from 2 independent experiments $*p \leq 0.05$ Kruskal Wallis test and Dunn's multiple comparison test. (B) Joint tissue from miR-34a$^{-/-}$ had significantly lower expression levels of *p19* mRNA compared with WT, at day 33 of induction of collagen-induced arthritis. WT (n=15) and miR-34a$^{-/-}$ (n=14) mice were sensitized according to the protocol described in Methods. Data are presented as dot-plots with median lines. $*p<0.05$, Mann-Whitney *u*-test.

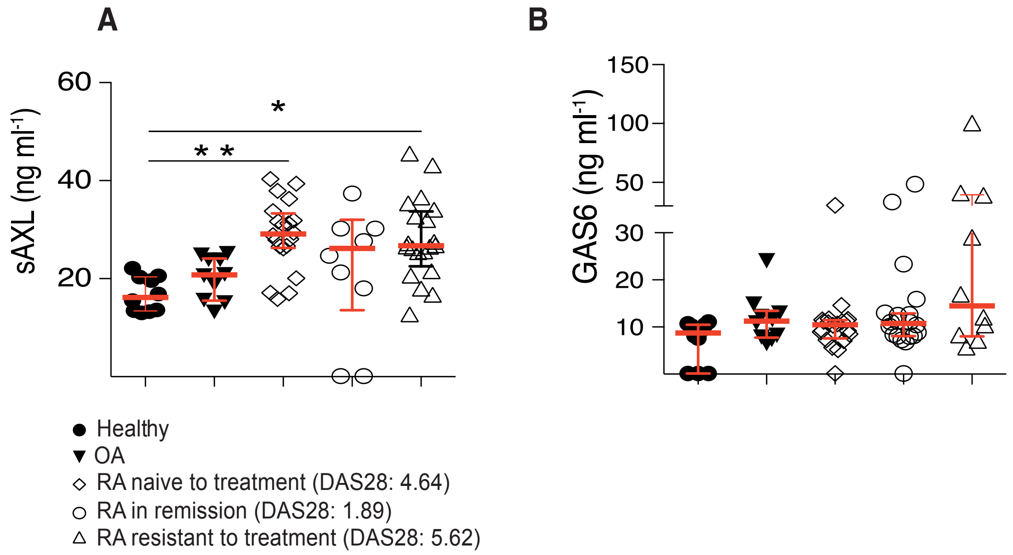

Supplementary Figure 10. The serum concentration of soluble (s)AXL is increased in RA patients. Soluble AXL (A) and GAS6 (B) were evaluated in sera of the study cohort described in Supplementary Table 3. Compared to healthy donors, sAXL was significantly increased in RA patients naïve to treatment (**p<0.001) and in RA patients resistant to treatment (*p<0.01), Kruskal Wallis test with Dunn's multiple comparison.

DAS28, disease activities score that includes 28 joints. RA Naïve to treatment = RA patients naïve to any Disease-Modifying Anti-Rheumatic Drugs (DMARDs); RA in remission = RA patients in stable remission achieved under combination therapy of Methotrexate + TNF inhibitor; RA resistant to treatment = RA patients not responding to conventional DMARDs/biologic treatments. The DAS scores in parentheses are the mean values for the groups.
